# Opportunities to enhance ward audit: a multi-site qualitative study

**DOI:** 10.1186/s12913-021-06239-0

**Published:** 2021-03-12

**Authors:** Michael Sykes, Richard Thomson, Niina Kolehmainen, Louise Allan, Tracy Finch

**Affiliations:** 1 Population Health Sciences Institute, Newcastle University, The Baddiley-Clark Building, Richardson Road, Newcastle upon Tyne, UK; 2 South Cloisters, University of Exeter, St Luke’s Campus, Heavitree Road, Exeter, UK; 3 Coach Lane Campus West, Northumbria University, Newcastle upon Tyne, UK

**Keywords:** Assurance, Audit and feedback, Hospital, Qualitative, Quality improvement

## Abstract

**Background:**

Hospitals in many countries are encouraged to develop audits to assess and improve the quality of care. Ward audit is a specific form of audit and feedback that is commonly used but little studied. The aim of this study is to describe the content and application of hospital ward audit in order to identify potential enhancements to such audits.

**Methods:**

Multiple qualitative methods were used to study a diversity sample of four English National Health Service organisations over a 16-month period. We undertook semi-structured interviews (*n* = 32), documentary analysis (*n* = 44) and 25 h of observations of healthcare workers involved in the design and implementation of ward audit. Data were analysed using framework analysis. Findings were presented iteratively to stakeholders who used them to develop a description of the content and delivery of ward audit.

**Results:**

Ward audit consisted of seven stages: impetus; method; preparation of staff; assessing practice; analysis; feedback; and decide on action to improve. Two key stages were the monthly assessment of practice using case note data extraction, and the resulting feedback to clinical staff, ward managers, matrons and directors of nursing. At three organisations, the case note data were extracted by staff and there was evidence that this resulted in misrepresentation of the clinical performance audited. The misrepresentation appeared to be associated with the anticipation of punitive feedback from directors of nursing and matrons, as well as time pressures and a lack clarity about the method of audit data collection. Punitive feedback was reported to occur if no data were collected, if data demonstrated poor performance or if performance did not improve.

**Conclusions:**

Organisations invest considerable clinical resources in ward audit, but such audits may have unintended, potentially negative, consequences due to the impacts from punitive feedback. We discuss potential enhancements to ward audit (e.g. providing feedback recipients with suggested actions for improvement) and discuss implications for theory. There is a need to reduce the use of punitive feedback.

**Supplementary Information:**

The online version contains supplementary material available at 10.1186/s12913-021-06239-0.

## Background

Globally, hospitals have developed internal quality assurance and improvement structures, of which audit and feedback is a substantial part [1–4]. Audit and feedback can be organised at different levels (e.g. nationally, regionally, by hospitals, teams or individuals). One widespread form of audit and feedback involves ward managers regularly receiving feedback about the clinical performance of staff on their ward. This audit measures the delivery of care (e.g. the prescribing and administration of medicines, the following of infection control procedures, the meeting of nutritional needs). Previous work to describe similar audits has used the terms ‘essence of care’ [5] and ward accreditation audits [6]. Within the current study, the term ‘ward audit’ was chosen as a generic way to describe this form of audit and feedback.

Audit and feedback can be an effective intervention to improve care, but its effectiveness varies (median absolute improvement 4.3%, interquartile range 0.5–16%) [7]. There is evidence and theory about what determines the effectiveness of audit and feedback. A systematic review of randomised controlled trials found that audit and feedback might be most effective when feedback is given repeatedly, provided by a supervisor or colleague, provided verbally and in writing, provided with solutions to sub-optimal performance, and when there is low baseline performance [7]. Theories and frameworks identify further potential influences (e.g. [8–10]) upon the effectiveness of audit and feedback. Brown and colleagues [10] synthesised evidence and theories related to feedback interventions to produce Clinical Performance Feedback Intervention Theory (CP-FIT). CP-FIT proposes that the response to feedback is affected by recipient factors (e.g. their beliefs about the feedback, and their knowledge and skills in quality improvement), feedback variables (e.g. the feedback goals, data collection and analysis methods, and the feedback display and delivery) and context (including organisation or team characteristics such as resources, priorities and leadership). CP-FIT describes multiple mechanisms, such as credibility and social influence, through which these factors operate to influence the feedback cycle and, as a result, clinical performance. Colquhoun and colleagues [11] interviewed experts in diverse theories related to audit and feedback and generated 313 theory-informed hypotheses of factors influencing whether audit and feedback leads to improvement (e.g. when steps are taken to prevent a defensive response to the feedback, and when accompanied by evidence supporting the behaviour change).

There have been calls to incorporate evidence and theory into the design of audit and feedback; to date much of this work has focused on national audits (e.g. [12]). The current study focused on a common form of audit developed at the organisation-level by hospital employees. We sought to describe the current content of ward audit, in order to identify potential evidence- and theory-informed enhancements.

## Method

This was a multi-site study using interviews, observations and documentary analysis, supported by two groups of stakeholders: a co-production group and an advisory group.

We studied four English National Health Service (NHS) acute hospital provider organisations, with approximately 4750 beds in total and with each organisation including between one and four hospitals (Table 1). Sites were purposively sampled for diversity; sampling involved identifying sites with different regulator assessment of effectiveness of care (Care Quality Commission rating from ‘requires improvement’ to ‘outstanding’) and selecting sites from each rating that varied in both size (ranging from 4000 to 15,000 full time equivalent staff) and performance on one of the key national audits (the National Audit of Dementia Care in General Hospitals) [13]. We initially sampled three organisations for diversity, the fourth organisation was sampled in order to test the description of the audit from the earlier sites. For this fourth site, we sampled a large hospital provider from a different geographical region that had a ‘requires improvement’ rating. The purposive sample of interviews, observations and documents (Appendix A) sought diverse perspectives upon the content and delivery of ward audit and was informed by documents reviewed, ongoing interviews, observations and stakeholder input, as described below.

**Table 1:** A description of the NHS organisations and sample

Site (NHS Organisation)	Regulator assessment (2014–2016)	Interviews	Observations	Documents
1	Requires improvement	9	9	24
2	Good	8	4	9
3	Outstanding	10	6	7
4	Requires improvement	5	0	4

### Interviews

The research team, supported by stakeholder involvement, identified potential healthcare professional participants according to their role. Potential participants were given information about the study by the hospital research department. All interviewees gave informed consent. During the semi-structured interviews, the interviewer (MS) asked participants to describe what happens during ward audit, exploring how, when, where and why it is done and involving whom [14]. The topic guide (Appendix B) explored participant’s involvement with the audit, their views on its effectiveness and whether there was anything they would change. The semi-structured nature enabled exploration of the elements most relevant to participants. During early interviews, the interviewer drew a diagram (Appendix C) derived from each participant’s descriptions and shared it with the participant for amendment during the interview. These were collated into a single diagram discussed with the co-production group and shared with later interviewees. Later interviewees were asked whether the diagram matched their experience. Their responses were used to refine and expand the description of what happens in ward audit.

### Observations

Written consent was sought for the observation of individuals. Where a group was observed, written, informed consent was sought from the senior person present, information was sent to those anticipated to be present (e.g. to meeting attendees) and posters were displayed giving information about the observations, the study and how to have data withdrawn from the study.

During observations, the researcher asked occasional questions in order to better understand what was being observed and to develop rapport with participants [15]. Observation data were recorded as field notes. Additional reflective notes were recorded after both the interviews and observations [16].

### Documentary analysis

Documentary analysis gathered data from secondary sources (e.g. minutes of meetings, policy documents) [17]. Documents (Appendix A) related to ward audit at the sites were accessed through the hospital research department, from participants and via the internet. The documents were pseudonymised and read in full. MS considered the position of the author, the intended audience and purpose of the document before seeking content that related to ward audit. The selected content was coded and analysed as described below.

The interviews and observations occurred during different site visits, over the same period as the documentary analysis (January 2018 to April 2019), thereby enabling each source of data to inform lines of enquiry.

### Stakeholder involvement

Work to describe the audit was supported by two stakeholder groups (Appendix D). A co-production group met nine times (18 h in total). Co-production group members were carers (*n* = 3), clinical leads (e.g. senior nurses or medical consultants) (n = 3) and organisational clinical audit leads (n = 3). During the first four co-production group meetings (8 h), MS facilitated small group work and whole group discussion to develop a description of the group’s pre-study understanding of audit and feedback. MS facilitated the co-production group to: inform the sampling of documents, and interview and observation participants; review analysed research data; consider the differences and similarities between the research data and their pre-study views; identify challenges to the analysis and interpretation of data; propose further avenues to explore; and iteratively develop the description. An advisory group included patient, academic, professional body, audit provider and commissioner input; it provided consultation support to the co-production group via MS. The researcher (MS) is a nurse who had recently managed organisation-level quality assurance in the NHS and had training and experience in qualitative methods and group facilitation.

### Analysis and synthesis

Interviews, observation field notes and reflective notes were transcribed, checked for accuracy and anonymised. Analysis involved familiarisation with the data followed by identifying initial themes within two interviews and two observations. MS compared and contrasted the themes across data sources and with the initial participant-described diagrams in order to create an initial framework. All transcripts were coded. Documents were pseudonymised, printed, coded and exemplar quotes extracted. Data were managed using Nvivo v12 (QSR International). MS analysed the data using inductive framework analysis [18], with over one hundred pages of samples co-coded by another researcher with extensive training and experience in qualitative methods (TF). Co-coding involved co-indexing and sorting, comparison of results and discussion to resolve disagreements. In addition, extensive quotes for each category and code were further challenged by all authors, and credibility increased through stakeholder challenge by the advisory and co-production group members. The themes were annotated onto the co-production group’s pre-study description and presented back to the co-production group. The group reviewed the annotations and identified challenges to the analysis and potential alternative sources of information. The process of data collection, analysis, annotation of the earlier description, presentation and adaptation was initially repeated twice. Prior to the third presentation back to the co-production group, the data were also considered against a framework for intervention description [19], the previous systematic review [7] and theory-informed hypotheses about audit and feedback [11]. This stimulated further questions in the co-production group, which resulted in a further iteration of data gathering, analysis and presentation. The framework was adapted throughout data collection to ensure all relevant information were included. The description from the eighth co-production group meeting was used to inform a topic guide for the fourth site where interviews and documentary analysis were undertaken. Following data from this fourth site, only minor alterations were made to the description in the ninth group meeting; a marker of theoretical saturation.

The study forms part of a larger project to describe and enhance audit and feedback in dementia care in acute hospitals that included a focus on both ward audit and the national audit of dementia [20].

### Findings

MS interviewed 32 healthcare staff, undertook 19 observations (25 h) and analysed 44 documents (Appendix A) from across the sites, as described in Table 1.

From the analysis, we identified that ward audit involved clinical performance data being presented to ward managers on a monthly basis. This included performance on: medicine management (e.g. the percentage of medicine charts where the administered medicines have were correctly recorded); infection control (e.g. the percentage of patient records with a visual infusion phlebitis score recorded in the last 24 h); nutrition (e.g. the percentage of patient records with a malnutrition universal screening tool completed within four hours of admission); bladder and bowel care (e.g. the percentage of patients with a urinary catheter who have catheter insertion date written in the records); and communication (e.g. the percentage of patients’ records with evidence that discharge has been discussed with carers or relatives).

**Fig. 1:**
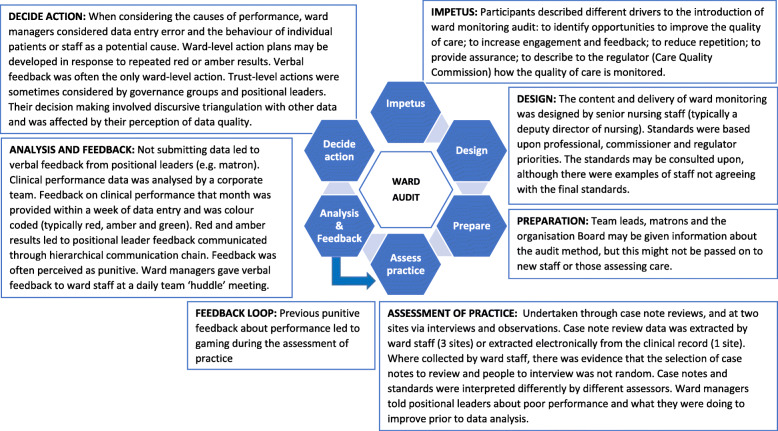
The stages of ward monitoring audit

We identified two high-level themes: audit stages and punitive feedback. There were seven different stages to the audit (Fig. 1): impetus; method; preparation of staff; assessing practice; analysis; feedback; and decide on action to improve. The stages were common across sites, with differences between sites within each stage, as described below. In addition to the stages, there was a cross-cutting theme of ‘punitive feedback’ which influenced how actors undertook the stages.

### Stages of ward audit

The *impetus* was described as being both from within and outside the organisation. Internal drivers were to assure and improve care:

“Why have we got a [ward audit] tool? It’s for the ward sister who owns that environment, to demonstrate where she thinks she’s up to in that environment. And that’s the most important thing for me. The results, I won’t say, don’t matter, but the results are secondary to that. I don’t like to see a sea of green, I’m much happier if there is some amber and red on there” (Interview 12, deputy director of nursing).

External impetus was to enable organisational managers to describe the monitoring of wards to the regulator:

Interviewer: “If you stopped doing [ward audit] tomorrow, what would be the impact?”

Matron: “The impact on patients?”

Interviewer: “On patients, on the staff, on the organisation.”

Matron: “I honestly don’t think that there would be an impact, directly, on patients. I don’t think it would change the quality of the documentation or the quality of care that is given to the patients.”

Interviewer: “Impact for staff?”

Matron: “Possibly relief, they’re not having to do it. For the organisation, I suppose it’s the accountability for giving that assurance to external organisations. How they’re monitoring the quality, they would have very little to back that up” (Interview 15).

The audit method was *designed* by directors of nursing or their deputies. Different organisations used different methods to gather data. One extracted audit data from the electronic record, the other three organisations used assessment of case notes by clinical staff. Two of the organisations also collected ward audit data through observations of practice, as well as staff and patient interviews.

In the *assessment of practice*, participants described that it was important to involve those providing care as it gave them feedback on the care they deliver; however, participants reported that self-assessment may affect data quality. The accuracy of data collection is described below. Study observations at one site found that completing five patient interviews and five case note reviews took over five hours, excluding data entry or staff interview data collection. Immediate clinical issues were identified (e.g. asking for help applying cream) during the assessment of practice. However, there was evidence that some clinical issues (e.g. a patient in the bed opposite asking for help; a patient repeatedly describing feeling cold at night) were not addressed.

The ward audit data *analysis* involved colour coding the results (e.g. red, amber, green). The targets used during the analysis may have been set higher than might be attained and were perceived as being aspirational (Interview 12, deputy director of nursing). The accuracy of red- or amber-rated performance was explored with the team manager; the accuracy of green (high) performance was not explored. Some actions were described as being beyond the control of the ward team (e.g. replacing wash basins).

The organisational board and directorates received summary data *feedback* giving collated performance by multiple wards (Interview 12, deputy director of nursing; Interview 20, clinical governance facilitator), whilst individual ward performance was sent to wards and matrons, directorate managers, directors of nursing and their deputies. The feedback did not include proposed solutions to address sub-optimal performance. At one organisation, ward-level comparison data were anonymised and presented bi-annually to ward managers.

Feedback to ward managers may not be new information, and this may affect whether it leads to action:

“As the manager, the matron, we know how we’re doing. I don't know if I really need this to know how I'm doing.” (Interview 28, ward manager)

Ward-level feedback was delivered verbally at a ward team meeting shortly after data were submitted and/or on receipt of analysed data. The verbal feedback was sometimes reiterated by email (Interview 21). Verbal feedback was repeated at daily huddle meetings of healthcare professionals, although this may depend upon the ward manager’s attendance at ward huddles (Observation 10, 12, 15, 16; Documents 25–31). Verbal feedback to ward staff may be the only response to poor performance:

“Sometimes all that’s needed is a butt kicking, if you know what I mean. A reminder that people need to get this done.” (Interview 10, Ward manager)

At two organisations, if a ward got consecutive red or amber results for the same standard, the matron (on behalf of the director of nursing) asked the ward manager to write an *action* plan. These may be escalated to positional leaders (Interview 17, Directorate manager, Organisation 1) or specialist teams. Actions may be documented in an action plan without anticipation of completion:

There was lots of stuff on it [the action plan] that you can’t [get]… Estates work, splashbacks that are stained that there’s no way on God’s good green earth I’m going to get. This is an old building. We’ve actioned it.” (Interview 10, ward manager).

Organisation- and directorate-level groups reviewed ward audit data to look for trends across wards (Interview 20, clinical audit facilitator). Specialist groups (e.g. medicine management group, pressure ulcer steering group) within the organisations may also review the data relevant to that group. In addition, the director or deputy director of nursing reviewed the data. They reported drawing upon other sources of information, including staff sickness, agency usage, patient acuity (Interview 4, directorate manager) and perceptions of team leadership (Interview 17, directorate manager) to assess whether and how to act. This triangulation was a reflective or discursive process (Interview 30, director of nursing).

Each of these stages took place within a wider organisational assurance and improvement system, proposed and designed to meet the needs of senior nursing leaders and external stakeholders.

“I think there’s been a desire in the last five or ten years to develop a tool that provides objective measurement of the quality of nursing care. I think most people have developed something internally that approximates the sorts of things I’ve described” (Interview 30, director of nursing)

“(Ward audit) is talked about as part of the (quality) account (for regulators and commissioners) … it’s on there. It doesn’t go into the detail, but it says if they want it, it’s available for them to talk about.” (Interview 17, directorate manager)

### The delivery and influence of punitive feedback

Punitiveness was a cross-cutting theme that provided an explanatory link between stages. The data described the reported experience and anticipation of punitive feedback and negative consequences by those receiving the feedback. Punitiveness largely related to the nature of verbal feedback described using words such as a ‘kick’ (interview 20, clinical governance facilitator), a ‘slap’ (Interview 30, Director of nursing), or a ‘whipping’ (Interview 10, ward manager). There were also non-verbal components, including additional scrutiny and senior management visits to the ward, which were described as intended, “to be persuasive or coercive. It depends on your viewpoint.” (Interview 10, ward manager).

**Fig. 2:**
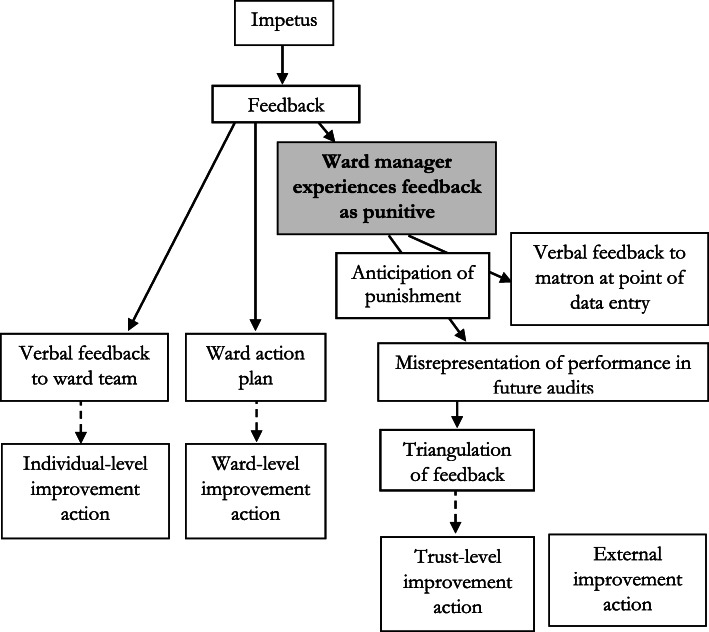
A representation of the influence of punitive feedback upon actions to improve care

Feedback from ward audit was reported to be punitive at three sites. There was evidence that this experience and anticipation of punitive feedback resulted in a range of unintended, potential consequences, (Fig. 2) as described below.

Evidence from one site describes that punitive feedback was replicated down a communication chain. The links in the chain were from the director of nursing, to matrons, to ward managers, to ward staff attending ward huddle meetings. Punitive feedback was described as happening in response to: submitting data describing red or amber rated performance; not submitting data; and not improving performance:

Ward manager: “They would be down on me like a tonne of bricks if I didn’t. If I hadn’t inputted the data, it would be, ‘Where’s your data? Why haven’t you done it?’ So, it’s just not worth not doing it.”

Interviewer: “Practically, what would that 'tonne of bricks' involve?”

Ward manager: “Oh, I would get…matron would be on my back. She’s really good, but somebody would be on her back. So, it would be like the house of cards that was toppled from the top and it would be all down. So, she would be under pressure and she would put that pressure on me to get that information where it should be, or some kind of explanation as to why it hadn’t been done. It’s just not worth it.” (Interview 10)

“that ward would just be targeted as a failing ward. It’s got a lot of work to do. It shouldn’t be that.” (Interview 15, matron)

The nature of the feedback may not be intentional. For example, senior nursing leaders described being aware of the feedback being experienced by recipients as punitive or inducing fear, and sought to balance this in their attempts to address complacency and produce performance data that they can, in turn, be described to the regulator:

“There is a balance, isn’t there? Between, and I’m not suggesting we’re trying to make people fearful because we’re absolutely not. But I think, for me, if I’m responsible for something, I need to know what standards are expected of me. But I want to know, if I can’t reach that standard, that someone will support me. I don’t want to be allowed to do what I want. We don’t want a complacent environment; we want people who know they have to work to achieve something.” (Interview 12, deputy director or nursing)

“We’re trying to reach standards right across the hospital and across the other hospitals that you’re providing the evidence to look at against, so there wouldn’t be anything [without ward audit]. And CQC (the regulator), when they come in, we’ve got something to show, ‘This is how we manage monitor, and manage- ‘, we’d have no evidence really.” (Interview 17, directorate manager)

Ward managers described both experiencing and anticipating punitive feedback from matrons and directors of nursing. The anticipation of this punitive feedback resulted in two main responses that sought to reduce such feedback. Firstly, ward managers provided their managers with information about actions being taken to improve sub-optimal care at the point of data entry (Interview 17, directorate manager). Secondly, the ward managers may seek to influence future performance by misrepresenting care.

Ward manager: “I have been known to go, “Yes” (when it should be no) I’m not going to lie to you.”

Interviewer: “How does that feel?”

Ward manager: “It feels like it’s a waste of my time. I’m ticking a box.” (Interview 10)

Misrepresentation may be related to fear of adverse comparative performance:

“I think they (ward managers) would be honest if everybody was scoring low. When it’s one out of… If everybody scored low then it wouldn’t be a problem, because then everybody has got some improvements to do.” (Interview 15, matron)

Hospital employees involved in the audit at different levels are aware that the results may not represent performance:

Matron: “I don’t think that they’re honestly completed. I think they’re meaningless. “

Interviewer: “And you know that because?”

Matron: “Because you review notes, you look at notes. Just from ward to ward, notes that come from another ward – you know, patient transfers. There is no way that places could be getting 100%. Often, a lot of places are getting 100% in many of the domains – or above 98% all of the time.” (Interview 15)

“(Clinical performance is) given the benefit of the doubt sometimes. I think it’s quite pedantic, the detail in the [ward audit] tool. So, if you’ve got 15 measures that tell you whether you get green on a chart, if one’s missing and you’re the ward sister, you might give green. Whereas if I’m an auditor, I probably won’t.” … “I think because it’s human nature. And if you ask someone to choose five sets of paperwork, which we do. Audit five sets of patient paperwork, that person might choose the worst five, they might choose the best five, and we have no control over that.” (Interview 12, deputy director of nursing)

“There is sometimes a concern of course that people lie, and they submit data which paints them in a very positive light.” (Interview 30, director of nursing)

Some ward managers are aware of the temptation to misrepresent care and delegate responsibility to others:

“sometimes it’s nice to put a junior member of staff doing it. They don’t realise what the consequences are.” (Interview 10, ward manager)

Misrepresentation led to the audit report demonstrating high performance which was repeatedly described as a “sea of green” (e.g. Interview 12, Deputy director of nursing). There was little evidence that the report stimulated organisation-level improvement actions, as represented by a dotted line in Fig. 2.

As described above, ward-level actions may be documented in an action plan, as a way to demonstrate a response to positional leaders, without anticipation of completion.

The ward manager may give ward staff feedback about actual performance but submit data to the corporate team that misrepresents performance:

“I sort of slap their wrists and tell them to wash their hands but really, I should mark them down then slap their wrist” (Interview 10, ward manager)

Directors of nursing, who triangulate the data with other sources in order to determine the need for action, may not view the findings as credible:

“You’ll see that the monthly return that I just showed you is effectively a sea of [perfect]. … I’m an inherent cynic so I tend to think that if it looks too perfect it probably isn’t.” (Interview 30, Director of nursing)

External stakeholders (commissioners and regulators) were told about the presence of ward audit, but not given the results. That ward audit data may not represent actual care might not be noticed by the external stakeholders (Interview 17, directorate manager), as a result Fig. 2 illustrates a lack of connection between the feedback and external stakeholder actions.

We found participants reported different reasons for the data being inaccurate. They reported that people may submit inaccurate data to save time and/or due to fear of scrutiny:

“I’m looking forward to a more mechanised tool which would reduce the audit burden, will release nurses to nurse, which is a good thing, but also might improve compliance and objectivity because it will be easier to do it properly and rather less easy to fake …. (to) enable them to be freed up to care for the patient directly.” (Interview 30, director of nursing)

Deputy director of nursing: “I think people just tick the box sometimes. They’re perhaps not doing the audits as thoroughly as they should.”

Interviewer: “Why not?”

Deputy director of nursing: “I don’t know really. Why would you not do it? Perhaps you want to be 100%, perhaps you’re frightened of the scrutiny it will bring you if you’re not 100%, perhaps it’s just the easiest thing to do.” (Interview 18)

Other positional leaders (e.g. directorate managers, deputy directors and directors of nursing) reported not being aware of how the feedback were perceived (Interview 30, director of nursing).

We found at least two organisational responses to address data quality. Firstly, there was evidence that organisations deliberately duplicated audits (Interview 12, deputy director of nursing), such that the same topic was assessed by different people through different audits (e.g. Observation 29). More recently, organisations have adopted additional approaches to address data quality; for example, one organisation was purchasing hand-held computers. The reported rationale for the computers was that they would be more reliable as it would give ward managers “fewer reasons for them to wish to cheat” because it would be quicker and data entry could be monitored (Interview 30, director of nursing). One organisation had developed, and one was developing, an accreditation approach to ward performance monitoring. This approach involved wards seeking recognition for the quality of care on the ward. Whether the ward achieved the status of a high-performing ward was based upon an assessment of care undertaken by the corporate nursing team, rather than ward staff. This may have altered ward managers’ willingness to report high performance:

“People have been hesitant to recommend their ward for assessment despite the fact that when they do the audit and when they report the audit they’re indicating compliance but they’re nervous because they think just that one day when somebody else comes to look at it there could be that variance.” (Interview 19, deputy director of nursing)

## Discussion

We explored ward audit at four diverse NHS organisations through interviews, observations and documentary analysis. We used inductive framework analysis, iteratively presenting the findings to stakeholders for challenge, to direct further data collection and to integrate the findings into a description of how audit was undertaken. We found that reporting the presence of ward audit to external stakeholders, notably the regulator (Care Quality Commission), was an important driver to positional leader support and organisational investment in the audit. The method of audit involved monthly data collection, typically by ward staff, in relation to topics including medicine management, infection control, nutrition, bladder and bowel care, and communication. We present the findings as a description of stages, before describing key findings regarding the nature of feedback and the accuracy of the data: Firstly, that feedback passing from more-senior to less-senior nurses was often perceived as punitive. Secondly, that ward collected data may not accurately represent the actual care provided. Furthermore, there was awareness at multiple levels that the results might not accurately represent clinical performance.

In discussing our findings, we compare the current content of ward audit to evidence [7] and theory [11], including CP-FIT [10] which was published after the data analysis. We present potential enhancements based upon this comparison. We then discuss potential adverse consequences from the current use of ward audit feedback and propose an intervention to address the use punitive feedback.

Our findings show that ward audit contains features consistent with audit and feedback evidence [7] and theory [10, 11]; evidence and theories also describe content which might enhance ward audit. Table 2 describes how current ward audit might be enhanced. For example, there is evidence and theory that presenting solutions to sub-optimal performance might make feedback more effective; as such, exploring influences upon performance and describing potential actions may support feedback recipients to improve care.

**Table 2:** Consistency with selected evidence and theory and potential enhancements

Findings consistent with selected evidence and theory	Findings different from selected evidence and theory	Potential evidence- and theory-informed enhancement
Feedback given by supervisor or peer [7] (e.g. ward manager to ward staff) Feedback given repeatedly [7] (e.g. daily at ward huddle meeting) The audit provides feedback to ward staff describing recent (this month’s) performance [10, 11], aside from issues of data accuracy. Feedback gains attention [11] Feedback to ward staff is given in person [11] Feedback to ward staff is given to a group [10, 11]	Recipients feel it is imposed upon them [10] Data is gathered by feedback recipients [10] The feedback is not perceived as accurate / credible [10, 11] Feedback describes team level, rather than individual, performance [10, 11] Feedback does not indicate trend over time [10] Feedback does not include solutions to sub-optimal performance [7, 10, 11] Feedback is perceived to punish sub-optimal performance [10]	Meaningful engagement of staff in the audit re-design Minimise data collection by automating and using alternative data sources, where possible Address impacts upon data quality, as discussed below. Individualise feedback Present performance over time Explore influences upon performance and describe how improvements can be made Explore and address causes of punitive feedback, as discussed below

Comparing our findings with CP-FIT [10] identifies similarities between our inductively developed findings and this more recent theory. We found that the design stage involved *goal setting*, assessment of practice involved *data collection* followed by *analysis* and *feedback.* Like CP-FIT, we describe unintended consequences where “health professionals may unethically manipulate data” (Brown et al., 2019); We also describe motivations for doing so.

We found potential extensions to CP-FIT: we describe the impetus stage, which whilst apparently similar to *goal setting*, related not to “the clinical topic and its associated clinical behaviours or patient” (Brown et al., 2019), but to the rationale for undertaking the audit. We found that this included the motivation to meet the regulator’s requirements. We found non-acceptance by senior managers of high performance, which differs from the non-acceptance of low performance by recipients described by Brown et al. We found different levels of action (patient, team, organisational and external), and describe differing responses by recipients at each of these levels. For example, triangulation at the organisational level demonstrated CP-FIT’s *verification* of all results, but only of ‘red’ results at the team-level.

In contrast to Brown et al., we found that the target (green-rated care) was important, as it was a proxy for the avoidance of anticipated punitive feedback. We describe the role of previous feedback upon data collection and ward managers’ anticipation of future feedback. Brown et al. state, “health professionals often had profound emotional reactions to feedback, both positive and negative…, we found no reliable evidence that these directly influenced the feedback cycle.”(p15). We describe the influence of the ward managers’ anticipation of punitive feedback, a subsequent emotional response (fear) and a behavioural response (misrepresentation of performance). We found that these responses impacted upon organisation-level leaders’ *verification perceptions, acceptance, intention* and *behaviour*. In CP-FIT, this influence may represent an additional *health professional variable* acting through *social influence, credibility* and *actionability* upon the feedback cycle. Articulating the influence of the previous experience of feedback upon pre-conceptions and subsequent actions provides a valuable extension to CP-FIT.

In addition to not incorporating best practices, our findings suggest that ward audit has potential negative impacts upon care. We propose two mechanisms by which the audit may adversely affect patient care: opportunity cost and an erosion of standards of care. There is considerable opportunity cost and evidence that staff prioritise data collection over direct patient care. For example, observations at one site suggested that the gathering of case note data took over five hours per month per ward and was done by ward managers or their deputies, with further time spent interviewing staff and entering data. There was some evidence that clinical issues were identified and addressed during this time (e.g. a patient requiring help to apply a cream), but there was also evidence that staff prioritised data collection over patient care (e.g. not going to a patient who requested help). Whilst purposive sampling undermines the drawing of economic conclusions, we propose that the time cost may exceed the benefits seen from some limited changes occurring in care as a result of the audit.

The requirement for nursing staff to spend time gathering ward audit data may have a more subtle effect: participants described ward audit as ‘a waste of my time’ and ‘meaningless’. We propose that the external prioritisation of perceived low value work may create feelings of discomfort in those being asked to prioritise time towards data collection for the audit. This discomfort may stem from being unable to meet personal goals relating to the delivery of high-quality care and externally set goals about the collection of data. It is possible that participants resolve this discomfort by downgrading their goals relating to standards of care [21]. Evidence that this might have happened comes from the finding that staff prioritised data collection over providing care to patients.

We found evidence that feedback was experienced as punitive. This supports earlier findings describing punitive perceptions of audit [22]. There is evidence [23] and theory [10, 24] that punitive feedback is less effective at improving care. We propose two mechanisms by which punitive feedback may affect patient care. Firstly, the experience of receiving punitive feedback may affect recipients’ mental health [25] and result in decreased staff satisfaction, which has been associated with increased staff turnover, reduced quality of care and lower patient satisfaction [24].

Secondly, we found that previous punitive feedback led to anticipated negative consequences which affected subsequent data quality. We found that the data was reported to be inaccurate and not credible. We propose that the lack of accuracy and credibility influenced commitment to change. Viewing the findings through the lens of clinical performance feedback intervention theory (CP-FIT), directors and deputy directors of nursing receive feedback through a table showing banded (red, amber, green) performance of teams. Their goal is to have high performance that provides assurance about the quality of care. They pay attention to red and amber performance, reflectively and discursively triangulating it with other data sources including acuity (patient population) and their perceptions of ward staffing and leadership (team characteristics). However, the subsequent organisation-level action response was mediated by a lack of accuracy (most results were ‘green’ and as a result no action plan was developed) and may have been affected by the reported lack of credibility, resulting in reduced commitment to change. Punitive feedback has been described as being less actionable at the individual level [23]. Our findings suggest that punitive feedback is also less actionable at the organisational level. The impact upon data quality and commitment to change highlights a potential concern for audits designed to be punitive (e.g .[26]). Reducing the use of punitive feedback may enhance the effectiveness of ward audit.

Future work should seek to implement enhancements to ward audit, including seeking to reduce the use of punitive feedback. Addressing punitive feedback may require consideration of the influences upon its use; for example, punitive feedback has been associated with leaders’ attributions that performance is related to individual, rather than system causes, or that performance is due to a lack of effort, rather than lack of ability or training [27]. An intervention targeting the attributions of those involved in the delivery of punitive feedback (e.g. directors of nursing, deputy directors of nursing, matrons, ward managers) may reduce recipient experience of punishment and increase the actionability of the feedback.

Reporting to the regulator was an important impetus to the ward audit. The regulator may also have a role in influencing the effectiveness of ward audit; for example, regulators could explore ward audit data quality through triangulation with other data, and/or by asking how ward audit stimulates organisation-level improvements based upon a review of influences upon performance.

One organisation had developed, and one was developing, accreditation mechanisms, whereby corporate nursing teams assess performance. Such accreditation audits have since been nationally recommended [28]. Our findings suggest that significant implementation work may be required to develop the collective leadership [28–30] and supportive culture [9] that would facilitate improvements from such accreditation systems. Current guidance [28] does not address this aspect of implementation. Another organisation in our study was investing further in electronic devices on which staff could record the data; this was described as a way to save time and potentially increase reliability. Future work could explore the impact of changes to data collection upon data accuracy.

### Strengths and limitations

We used multiple methods and triangulated data to explore ward audit at diverse NHS organisations. The findings were presented to a stakeholder group for challenge, to identify where further data were needed and to synthesise the description. As part of this work to enhance the credibility of the findings, the research team and stakeholder group considered emergent findings against a framework for intervention description, and against previous evidence and theory.

We do not provide identifiers as to which findings came from the which organisation in order to protect the anonymity of participants. The fourth site was sampled to test the description based upon data from earlier sites, and had the same CQC rating as an earlier site. We did not sample hospitals rated as ‘inadequate’; hospitals with this rating may have used different approaches to ward audit from the sites we studied. We describe reported perceptions of feedback. The nature of feedback beyond ward level was not observed and it may be that ward managers feel that the data profiles their personal performance, and that this makes feedback feel punitive [31]. However, the repeated description, and the observed and reported impact upon the assessment of practice, support the findings we report. Additionally, that feedback was perceived as punitive may be more important than an assessment of its actual content and delivery.

We describe a communication chain at one site from director of nursing to ward staff. It is possible that there are other links in this chain. No assumption is made as to the intention or source of the nature of the feedback. In particular, it may be that the delivery, experience or anticipation of punitive feedback reflects organisational norms regarding attributions or patterns of communication; it may also reflect external influence [32].

## Conclusions

Organisations invest considerable resources in ward audit. We highlight evidence and theory-informed enhancements to ward audit. In particular, there is evidence that at some organisations the data are reported to be unreliable, that this is recognised by those involved and that data accuracy is associated with feedback being perceived as punitive. We recommend that recipients of ward audit data explore the reliability of the data and consider the potential role of punitive feedback as a factor undermining data quality and subsequent quality improvement. We propose national and organisation-level work to enhance ward audit, including through the implementation of non-punitive feedback.

## Supplementary Information

**Additional file 1:** **Appendix A**: The study sample (Note: Titles standardised in order to maintain anonymity).

**Additional file 2: Appendix B**: Interview topic guide v3.

**Additional file 3: Appendix C**: Example interview diagram.

**Additional file 4: Appendix D**: A diagrammatic representation of study data sources.

## Abbreviations

CP-FIT: Clinical Performance Feedback Intervention Theory
CQC: Care Quality Commission
NHS: National Health Service
MS: Michael Sykes
TF: Tracy Finch
