# Supplementary material for: Opportunities to enhance ward audit: a multi-site qualitative study
Source: BMC Health Serv Res. 2021 Mar 12;21:226. doi: 10.1186/s12913-021-06239-0 (PMC7971099; doi:10.1186/s12913-021-06239-0)
Supplement: Supplementary file 1 — Additional file 1: Appendix A: The study sample (Note: Titles standardised in order to maintain anonymity). [file 12913_2021_6239_MOESM1_ESM.docx]

**APPENDIX A:** The study sample (Note: Titles standardised in order to maintain anonymity)

| **Interviews:**  1 = Clinical audit lead  2 = Clinical audit administrator  3 = Consultant audit lead  4 = Directorate nurse manager  5 = Deputy director of nursing  6 = Dementia nurse specialist  7 = Ward manager  8 = Deputy director of nursing  9 = Clinical audit administrator  10 = Ward manager  11 = Improvement project lead  12 = Deputy director of nursing  13 = Clinical audit facilitator  14 = Dementia nurse specialist  15 = Matron  16 = Directorate manager | 17 = Directorate manager  18 = Deputy director of nursing  19 = Deputy director of nursing  20 = Clinical governance facilitator  21 = Staff nurse  22 = Pharmacist  23 = Staff nurse  24 = Nurse consultant  25 = Occupational Therapist  26 = Head of clinical specialty  27 = Dementia audit lead  28 = Ward manager  29 = Clinical audit facilitator  30 = Executive Director of nursing  31 = Deputy Director of nursing  32 = Matron |
| --- | --- |

| **Observations**  1 = Clinical effectiveness committee (Trust level meeting held in Board room)  2 = Dementia steering group (Meeting chaired by consultant to discuss improvements in dementia care)  3 = Clinical governance committee (Trust level meeting that reports to the Trust Board)  4 = National audit preparation meeting (Meeting between dementia nurse specialist, Trust quality assurance lead and clinical audit lead to plan the data collection)  5 = Clinical governance meeting (Trust level meeting.)  6 = Dementia steering group (Meeting chaired by consultant to discuss improvements in dementia care)  7 = Dementia steering group (Meeting chaired by consultant to discuss improvements in dementia care)  8 = Record review (Data collection)  9= Record review (Data collection)  10 = Ward meeting (Multidisciplinary huddle meeting to discuss both patient care and more general issues)  11 = Directorate governance meeting (Specialty quality assurance meeting that reports to Trust level committee)  12 = Ward meeting (Multidisciplinary huddle meeting to discuss both patient care and more general issues)  13 = Directorate governance meeting (Specialty quality assurance meeting that reports to Trust level committee)  14= Trust Clinical Effectiveness meeting (Trust level meeting held in Board room.)  15 = Ward meeting (Multidisciplinary huddle meeting to discuss both patient care and more general issues)  16 = Ward meeting (Multidisciplinary huddle meeting to discuss both patient care and more general issues)  17 = Record review (Data collection)  18 = Clinical governance meeting (Trust level meeting that reports to the Trust Board.)  19 = Clinical audit project meeting (Project meeting to discuss set up of new audit process) |
| --- |

| **Documents:**  1 = Quality Accounts (a publicly available report about the quality of services published each year by NHS healthcare provider)  2 = Quality Accounts  3 = Quality Accounts  4 = Care quality commission report (a publicly available report about the quality of services published by the regulator)  5 = Care quality commission report  6 = Care quality commission report  7 = Quality Strategy (a document describing actions to improve care)  8 = Quality Strategy  9 = Quality Strategy  10 = Clinical audit policy  11 = Specialty Governance Group Action log  12 = Clinical effectiveness committee terms of reference  13 = Clinical effectiveness paper (a report to the clinical effectiveness committee)  14 = Clinical audit policy  15 = Ward audit tool (Data collection form)  16 = Ward audit tool  17 = Ward audit tool  18 = Ward audit tool  19 = Clinical effectiveness paper  20 = Specialty governance minutes (minutes from a specialty-level meeting to consider assurance of quality)  21 = Specialty governance minutes  22 = Specialty governance minutes  23 = Governance paper providing update on audit within the specialty  24 = Trust board papers (publicly available minutes from the most senior Trust Board meeting)  25 = Ward huddle checklist  26 = Ward huddle checklist  27 = Ward huddle checklist  28 = Ward huddle checklist  29 = Ward huddle checklist  30 = Ward huddle checklist  31 = Ward huddle checklist  32 = Clinical audit handbook (a training resource produced within one site)  33 = Trust newsletter (written information for staff about the quality of care)  34 = Trust newsletter  35 = Trust newsletter  36 = Quality strategy  37 = Specialty governance minutes  38 = Specialty governance minutes  39 = Ward audit tool  40 = Clinical audit policy  41 = Specialty action log  42 = Clinical governance minutes  43 = Ward audit tool  44 = Care quality commission report |
| --- |
