# Supplementary material for: Opportunities to enhance ward audit: a multi-site qualitative study
Source: BMC Health Serv Res. 2021 Mar 12;21:226. doi: 10.1186/s12913-021-06239-0 (PMC7971099; doi:10.1186/s12913-021-06239-0)
Supplement: Supplementary file 2 — Additional file 2: Appendix B: Interview topic guide v3. [file 12913_2021_6239_MOESM2_ESM.docx]

**Appendix B: Interview topic guide v3**

1. Could you describe your role?
2. What do you understand by the term audit and feedback? (Can prompt that often called ‘clinical audit’)
3. Different types of audits have been described.
   1. Do you recognise these types? (Show list from earlier data)
   2. Which of these types are you involved with?
   3. How do they differ?
4. Do you come into contact with ward audit? What is your involvement with that audit?
5. The ward audit has been described like this (Show collated diagram from earlier data). Does this match your experience? With which parts of this do you get involved?
6. Explore each part^i^:
   1. When does it happen?
   2. Where does it happen?
   3. Who is involved?
   4. How is it done? Is it always done like that? (If appropriate, prompt about materials involved)
   5. How does that feel? How do other people feel about that?
   6. Which documents and/or potential observations/interview participants could provide more information about this part?
7. Audit is used for different reasons, why do you think it is used here? Any other reasons?
8. Some people use audit to improve care. What do you think about that?
   1. How much do you think it improves care?
   2. How does it improve care?
   3. What affects whether it improves care? (Use collated diagram as prompt)
9. If you could change anything about ward audit, what would you change?
10. What would happen if the hospital didn’t do ward audit? (Can prompt: Would things be different, and if so, how?)
11. Is there anything else you would like to add?

Thank you

Footnote:

^I^ Interviewer has a personal version of the collated diagram with stage and participant specific questions based upon emergent findings and stakeholder feedback
