# Supplementary figures and images for: Opportunities to enhance ward audit: a multi-site qualitative study
Source: BMC Health Serv Res. 2021 Mar 12;21:226. doi: 10.1186/s12913-021-06239-0 (PMC7971099; doi:10.1186/s12913-021-06239-0)

**Appendix C:** Example interview diagram


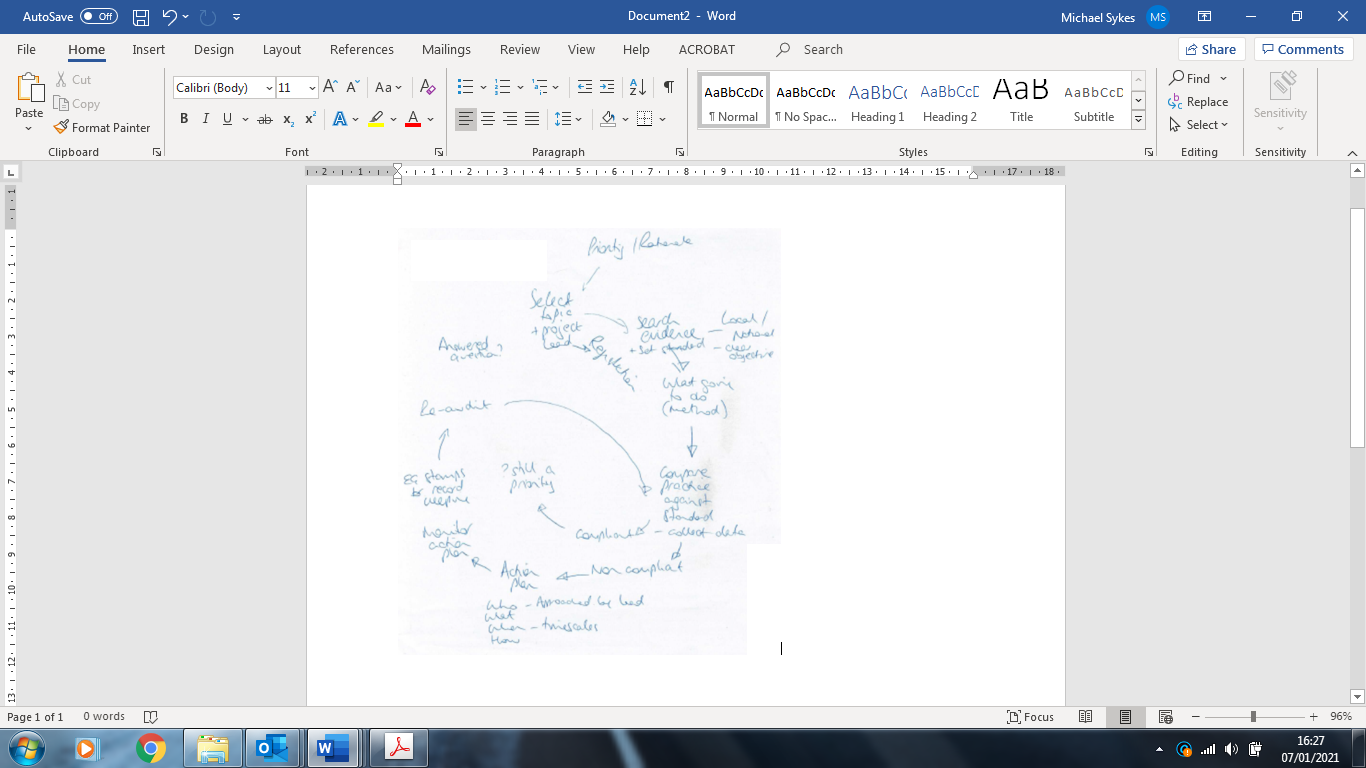

Supplement: Supplementary file 3 — Additional file 3: Appendix C: Example interview diagram. [file 12913_2021_6239_MOESM3_ESM.docx]
