# Supplementary material for: Opportunities to enhance ward audit: a multi-site qualitative study
Source: BMC Health Serv Res. 2021 Mar 12;21:226. doi: 10.1186/s12913-021-06239-0 (PMC7971099; doi:10.1186/s12913-021-06239-0)
Supplement: Supplementary file 4 — Additional file 4: Appendix D: A diagrammatic representation of study data sources. [file 12913_2021_6239_MOESM4_ESM.docx]

**Appendix D:** A diagrammatic representation of study data sources

**Describe ward audit**

**Identify potential enhancements**

Stakeholder involvement: Co-production group (9 meetings; 18 hours); Advisory group input

Ivers et al (2012)

Ivers et al (2012)

Colquhoun et al (2017)

Colquhoun et al (2017)

Hoffmann et al (2014)

Primary data from interviews (n=32), observations (n=19) and documentary analysis (n=44)

Secondary data from previous systematic review

Theory

Secondary data from previous systematic review

Specifying framework

Co-production group pre-study views

Theory

Theory

Brown et al (2019)

Research team
